# Association between dietary patterns and subjective and objective measures of physical activity among Japanese adults aged 85 years and older: a cross-sectional study

**DOI:** 10.1017/S0007114522003993

**Published:** 2023-09-28

**Authors:** Tao Yu, Yuko Oguma, Keiko Asakura, Yukiko Abe, Yasumichi Arai

**Affiliations:** 1 Graduate School of Health Management, Keio University, 4411 Endo, Fujisawa City 252-0883, Japan; 2 Sports Medicine Research Center, Keio University, 4-1-1Hiyoshi, Kouhoku-ku, Yokohama City 223-8251, Japan; 3 Department of Environmental and Occupational Health, School of Medicine, Toho University, 5-21-16 Oomori nishi, Oota-ku 143-8540, Japan; 4 Center for Supercentenarian Medical Research, Keio University, 35 Shinanomachi, Shinjuku-ku 160-8582, Japan; 5 Keio University Faculty of Nursing and Medical Care, 4411 Endo, Fujisawa City 252-0883, Japan

**Keywords:** Ageing population, Healthy behaviour, Dietary pattern, Physical activity, Healthy life expectancy

## Abstract

A healthy diet and regular physical activity (PA) are delineated as healthy behaviours. Their implementation is associated with better health outcomes and improved quality of life. There is less evidence of a relationship between dietary patterns (DP) and PA, especially in adults aged ≥ 85. Hence, this cross-sectional study investigates the association between DP and PA in people of this age group, using the data from The Kawasaki Aging and Well-Being Project. Brief-type self-administered diet history questionnaire was used to estimate the intake of fifty-eight types of food. After energy adjustment, principal component analysis was performed to identify DP. PA was measured objectively using an accelerometer and subjectively using a questionnaire validated for this age group. Thousand participants (median age: 86·9 years, men: 49·9 %) were included in the analysis. Three major DP (DP1 ‘various foods’, DP2 ‘red meats and coffee’, DP3 ‘bread and processed meats’) were identified. DP1 ‘various foods’ was similar to DP previously named ‘healthy’ or ‘prudent’ and showed a positive association with PA time (PAT) as measured by accelerometer (B, 6·25; 95 % CI 0·13, 12·37) and relatively shorter sedentary behaviour (SB) time. DP2 ‘red meats and coffee’ and DP3 ‘bread and processed meats’ were negatively associated with PAT and positively associated with SB time. This study observed the relationship between diet and PA behaviours in adults aged ≥ 85, with healthier and more food-diverse DP associated with longer PAT and relatively unhealthy DP with shorter PAT.

Diet and physical activity (PA) are closely related to energy intake and expenditure behaviours, known as energy balance-related behaviours (EBRB)^(1)^. Consuming nutritious food and doing more PA, a part of healthy behaviours (HB), are associated with health-related outcomes (e.g., quality of life, cognitive function, disease development and functional impairment) in populations of various age and socio-economic groups^(2–8)^. Diet is not meant to evaluate the intake of any one nutrient but rather the total food intake consisting of different nutrients. Research using the concept of dietary patterns (DP) has certain advantages, including the fact that in reality, humans do not eat food with a single nutrient but rather consume food that includes multiple nutrients^(9,10)^. DP can be identified using either ‘a priori approach’ or ‘a posteriori approach’^(11)^. In former approach, the quality of a population’s diet is evaluated by assessing adherence using measures such as the Mediterranean diet score and Dietary Approaches to Stop Hypertension index^(12)^. The latter approach uses factor analysis or principal component analysis (PCA) to identify specific DP in an observed population, with most studies identifying major DP from each data and examining their association with PA, and health-related outcomes such as physical performance^(13,14)^. PA is essential for maintaining physical and mental health. It improves immunity against various diseases. Also, potential health demerits of sedentary behaviour (SB) have also been reported^(15–18)^. The mechanisms linking PA/SB with health-related outcomes are not fully understood. Still, it has been suggested that PA and SB may act oppositely to the body’s inflammatory state but by different mechanisms^(19)^. With estimates that 4–5 million deaths per year can be avoided if the world population were more active, the WHO has issued guidelines for PA and SB in 2020^(20)^. Globally, the population aged 65 and over is growing faster than all other age groups, with an increasing number of older adults (≥ 85 years), particularly in Asian countries like Japan and China (especially Hong Kong). Under these circumstances, the critical issue is extending the period during which people are independently active, that is, healthy life expectancy^(21)^. Improved healthy life expectancy has been much discussed from both exercise and diet perspectives, and public health interventions targeting PA and healthy diets are known to be cost-effective^(22)^. However, their relationship is not explored much, especially in older adults. A key reason for this is that previous studies have focused on adults aged ≤ 65 years or older adults (generally around aged 65–70 years), and considerably less research has been conducted on adults aged ≥ 85^(23,24)^. In addition, PA measurements in many studies were performed using self-administered questionnaires, which had several limitations, including recall bias of participants and over-or-underestimation of the amount of PA^(23)^.

Examining the association between DP and PA in adults aged ≥ 85 would help in understanding their lifestyle behaviours. Both DP and PA play an important role in influencing the health of older people, thus clearing up the association between them is important for intervention research for older people and evidence-based policy-making and is a necessary component of successful ageing in a super ageing society^(21,25)^. Therefore, in this study, we aimed to determine the relationship between DP and PA in older adults aged ≥ 85 after identifying the DP and measuring PA using questionnaires and an accelerometer.

## Methods

### Study population

**Fig. 1.**
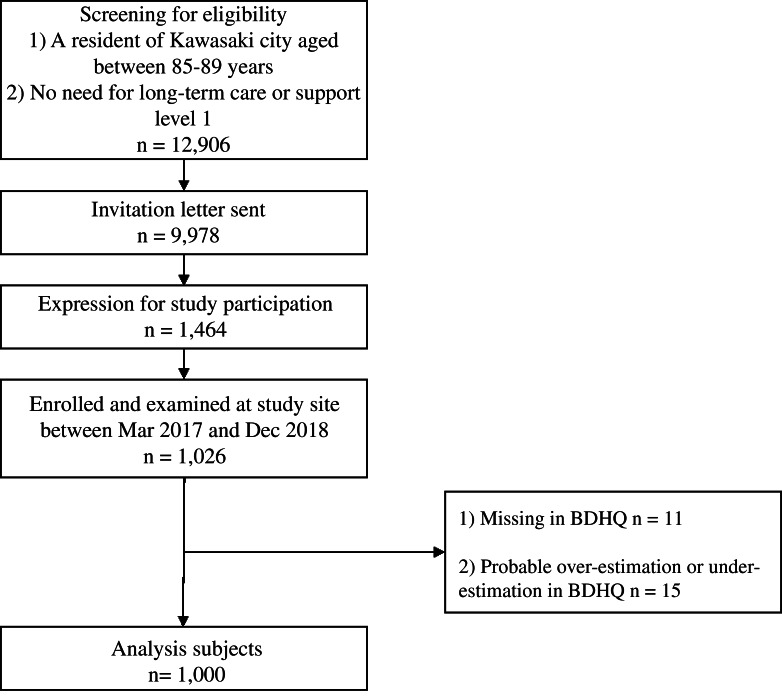
Recruitment of participants.

This cross-sectional study was conducted using data from The Kawasaki Aging and Well-Being Project (KAWP) in Kawasaki City (Kanagawa Prefecture, Japan). KAWP inclusion criteria were as follows: (1) residents of Kawasaki City located in the Tokyo metropolitan area; (2) aged 85–89 years; (3) needing nursing care or up to support level 1 (no limitation in basic activities of daily living (ADL)) and (4) ability to visit the study sites (several hospitals in Kawasaki City) independently. Using the Basic Resident Ledger and the long-term care insurance database, 12 906 residents were screened as potential participants; 9978 residents were mailed invitations to participate in this study and 1464 eligible residents indicated their willingness to participate in the study between March 2017 and December 2018. One thousand and twenty-six independent older adults individuals were enrolled in the KAWP and underwent a comprehensive baseline assessment of physical, mental and cognitive performance and social participation (Fig. 1)^(26)^. Those with missing dietary surveys (*n* 11) and large errors in dietary survey reporting (estimated energy intake > 16736 kJ (4000 kcal) or < 2510.4 kJ(600 kcal), *n* 15) were excluded.

The KAWP was approved by the Ethics Committee of Keio University School of Medicine (ID: 20160297) and registered in the University Hospital Medical Information Network Clinical Trials Registry (ID: UMIN000026053, Registered 08 February 2017). Participation in the KAWP and the survey were in accordance with the Declaration of Helsinki, and written informed consent was obtained from the participants who were interested in the survey after thorough explanation. These participants could withdraw their consent at any time upon request.

### Identification of dietary pattern

The dietary survey used the brief-type self-administered diet history questionnaire, validated with 3-d semi-weighed dietary records in the studied age group^(27)^. Though the participants filled the brief-type self-administered diet history questionnaire themselves, they were later interviewed, and the brief-type self-administered diet history questionnaire was modified as needed by a trained researcher. The brief-type self-administered diet history questionnaire was used to determine the type of meals consumed in the past month. It estimated nutrient intake based on the type, amount and frequency of foods. This study identified the major DP of the study population using the PCA, which is the posteriori DP, suitable for identifying DP in the target population. Before identifying DP, we classified fifty-eight foods into thirty-three foods/food groups, based on a previous study of Japanese aged 70–90 years^(28)^. There are two methods for energy adjustment: the residual method and the density method, but as the residual method is strongly dependent on the population values and cannot be used for individual data, the density method was used for this study^(29)^. After energy adjustment, PCA was performed, based on the eigenvalues and scree plot, three principal components of nutritional significance could be identified and a principal component score was calculated. The principal component scores were continuous variables ranging from –1 to 1. Higher scores indicate greater adherence to DP.

### Measurement of physical activity

PA was measured using a questionnaire-based subjective survey and an accelerometer-based objective measurement, and Metabolic equivalents (MET) are often used to measure the intensity of PA^(17)^. The questionnaire used the modified Zutphen physical activity questionnaire, which was validated by a similar age group^(30)^. Physical activity questionnaire is based on the walking speed (fast, normal and slow), walking time and activity intensity for each time and type of exercise (e.g. callisthenics and resistance training) and is multiplied by the number of hours per day and number of times per week to calculate the amount of PA (MET × h/week). PA index (PAI, sum of walking and exercise, MET × h/week), PA time (PAT, sum of walking and exercise time, min/d) and SB time (SBT, such as TV viewing time, min/d) were used in this study. Objective measurements of PA were recorded using a validated device (Active style Pro, HJA-750C, Omron Healthcare Corporation) with a built-in triaxial accelerometer. The participants were asked to wear the device continuously for 1 week, at all times during the day except when taking a bath and sleeping at night. The absence of acceleration signal detection for longer than 60 consecutive minutes was defined as ‘non-wear’, and valid data were considered for wearing the device for at least 10 h/d for at least 3 d (participated in the accelerometer survey but with insufficient days, *n* 57). The intensity of accelerometer measurements of 1·5 MET or less was regarded as SB, and 1·6 MET or more was considered PA^(17)^. PAI (MET × h/week), PAT (min/d) and SBT (min/d) were set as outcomes.

### Assessment of covariates

Participants’ demographics, including age, sex, ADL (as assessed by the Barthel Index), BMI (calculated as weight in kilograms divided by height in metres squared (kg/m^2^)), WHO-Five well-being index (WHO-5), socio-economic factors, such as years of education, economic status and living status, and lifestyle habits, such as smoking were also examined. Additional medical history (heart disease, kidney disease, hypertension, diabetes, dyslipidaemia and cancer) and need for long-term care were also identified. Cognitive function was assessed by a professional psychologist in a private room using Mini-Mental State Examination (MMSE).

### Statistical analysis

Quantitative variables (such as age or BMI) were reported as medians (25th–75th percentile), and categorical variables (such as sex or living status) were reported as the number and percentage (%) in each category. Mann–Whitney U test and Chi-square test (or Fisher’s exact test) were used to compare the groups. A linear regression model was used to examine the association between DP and PA. For PA with outcomes measured subjectively, in addition to principal component scores, age, sex, BMI and MMSE were adjusted in Model 1. Model 2 was adjusted for ADL, WHO-5, years of education, living alone, economic status, smoking habits, medical history and need for long-term care in addition to the variables in Model 1. For PA measured objectively, in addition to principal component scores, age, sex, BMI, MMSE and wearing time were adjusted in Model 1. Model 2 was adjusted for ADL, WHO-5, years of education, living status, economic status, smoking habits, medical history and need for long-term care in addition to the variables in Model 1. The data were analysed using SPSS version 26.0 (IBM Japan). Statistical significance was defined as *P* < 0·05.

## Results

Table 1 provides details on the DP identified in this study. PCA identified three major DP. The first, DP1, was characterised by the intake of a variety of plant foods, including green and yellow vegetables and other vegetables, and was also positively loaded with fish and seafood intake; thus, it was named ‘various foods’. The second, DP2 ‘red meats and coffee’, was characterised by the intake of foods considered rich in protein, of which red meat (pork and beef) had the highest loading. In addition, coffee also showed a high loading, so it was named ‘red meats and coffee’. The third, DP3, was characterised by intake of bread and processed meat (such as ham and sausage) and was named ‘bread and processed meats’. The three DP accounted for 10·7, 6·5 and 5·7 % of the variance, respectively.

**Table 1.** Identification of dietary patterns (DP)

	DP1 Various foods	DP2 Red meats and coffee	DP3 Bread and processed meats
Cooked rice	–0·36		–0·63
Noodles			
Bread	–0·20		0·49
Miso soup			–0·51
High fat milk			
Low fat milk			
Red meats		0·28	
Chicken		0·20	
Processed meats		0·22	0·36
Fish	0·33		–0·26
Shellfish			
Seafood	0·22		–0·29
Egg		0·23	
Potatoes	0·43		
Soya products	0·38	0·25	–0·26
Green and dark yellow vegetables	0·67		
Other vegetables	0·69	0·22	
Pickled vegetables	0·40		
Salad vegetables	0·53	0·25	0·22
Mushrooms	0·51		
Seaweeds	0·50		
Fruit	0·35		
Confectioneries		–0·60	0·28
Ice cream		–0·34	
Sugar	–0·25	0·62	0·23
Fat and oils	0·29	0·28	0·33
Alcoholic beverages	–0·24		
Green tea			–0·21
Black and oolong tea			0·21
Coffee	–0·28	0·45	0·39
Soft drinks			0·27
Fruit and vegetable juice			0·22
Seasonings		0·66	–0·22
Total	3·52	2·15	1·89
Initial eigenvalues % of variance	10·66	6·51	5·72
Cumulative %	10·66	17·18	22·90

Numbers indicate the loading each food group or food accounts for, and items with an absolute value <0.20 are left blank.

Table 2 shows the characteristics of the participants and the DP in this study. The study included 499 men (49·9 %), with median age, BMI, MMSE and ADL of 86·9 years, 23·1 (kg/m^2^), 26 and 100, respectively. In the DP1 ‘various foods’, there were significant differences in sex, MMSE, WHO-5, smoking habits and economic status between the low and high-trend groups (meaning below or above the median on the principal component scores). In the DP2 ‘red meats and coffee’, differences in the proportion working were identified, with a significantly smaller proportion in the high-trend group. In the DP3 ‘bread and processed meats’, significant differences were observed in sex and years of education, with more women and longer years of education in the high-trend group.

**Table 2.** All participants and each dietary pattern (DP)’s characteristics (Numbers and percentages; medians and percentiles)

			DP1 Various foods					DP2 Red meats and coffee					DP3 Bread and processed meats				
Variables	All participants		Low-trend group		High-trend group		*P*	Low-trend group		High-trend group		*P*	Low-trend group		High-trend group		*P*
	*n*	%	*n*	%	*n*	%		*n*	%	*n*	%		*n*	%	*n*	%	
Men	499	49·9	296	59·2	203	40·6	<0·001	241	48·2	258	51·6	0·31	268	53·6	231	46·2	0·02
	Median	25th–75th percentile	Median	25th–75th percentile	Median	25th–75th percentile		Median	25th–75th percentile	Median	25th–75th percentile		Median	25th–75th percentile	Median	25th–75th percentile	
Age	86·9	85·9–88·2	86·8	85·9–88·2	86·9	85·9–88·2	0·88	86·9	85·9–88·2	86·8	85·9–88·2	0·60	86·8	85·8–88·1	86·9	85·9–88·2	0·30
BMI	23·1	21·1–25·1	23·1	21·1–25·1	23·0	21·1–25·2	0·67	23·1	21·2–25·3	23·1	21·0–25·0	0·59	23·1	21·2–25·2	23·0	21·1–25·1	0·44
Mini-Mental State Examination	26	24–28	26	24–28	27	25–29	<0·01	26	24–28	26	24–28	0·95	26	24–28	26	24–29	0·31
WHO-Five well-being index (*n* 980)	19	15–22	18	15–21	20	16–23	<0·001	20	15–22	19	15–22	0·24	19	15–22	20	15–22	0·71
Activities of daily living (*n* 997)	100	100–100	100	100–100	100	100–100	0·13	100	100–100	100	100–100	0·52	100	100–100	100	100–100	0·83
Year of education	11	9–14	11	8–14	11	9–14	0·39	11	9–14	11	9–13	0·76	10	8–13	12	10–14	<0·001
	*n*	%	*n*	%	*n*	%		*n*	%	*n*	%		*n*	%	*n*	%	
Living alone (*n* 983)	277	28·2	146	29·7	131	26·7	0·32	135	27·4	142	29·0	0·62	128	26·0	149	30·3	0·14
Smoking habit (*n* 997)																	
Smoker	42	4·2	30	6·0	12	2·4	<0·001	19	3·8	23	4·6	0·76	22	4·4	20	4·0	0·91
Ex-smoker	375	37·6	212	42·5	163	32·7		185	37·1	190	38·1		189	38·0	186	37·3	
Non-smoker	580	58·2	257	51·5	323	64·9		294	59·0	286	57·3		287	57·6	293	58·7	
Economic status (*n* 974)																	
Very good/good	590	60·6	275	56·6	315	64·5	0·04	302	62·0	288	59·1	0·44	291	60·0	299	61·1	0·78
Neither	200	20·5	110	22·6	90	18·4		92	18·9	108	22·2		104	21·4	96	19·6	
Bad/very bad	184	18·9	101	20·8	83	17·0		93	19·1	91	18·7		90	18·6	92	19·2	
Working (*n* 988)	141	14·3	73	14·7	68	13·8	0·72	91	18·3	50	10·2	<0·001	68	13·8	73	14·7	0·72
No disease history (*n* 963)	143	14·9	67	13·8	76	15·9	0·49	71	14·8	72	14·9	0·48	72	14·9	71	14·8	0·72
Need for long-term care (*n* 977)	113	11·6	60	12·3	53	10·9	0·55	56	11·4	57	11·7	0·92	48	9·8	65	13·4	0·09

The effective number of participants is shown next to the item; values are shown as median (25th-75th percentile) or number (%), the p-value is a test of the difference between the high and low trend groups for each dietary pattern; a low or high trend group means Scoring lower or higher than the median on the principal component score.Body mass index is calculated by kg/m², Barthel Index evaluates activities of daily living, and disease history includes heart disease, kidney disease, cancer, hypertension, diabetes, and dyslipidemia.

Table 3 shows the PA status of all participants and by DP. According to the results of the questionnaires, the median PAI, PAT and SBT were 15·9 MET × h/week, 190·0 min/d and 2·5 h/d, respectively. As measured by the accelerometer, PAI, PAT and SBT median values were 21·1 MET × h/week, 309·1 min/d and 534·3 min/d, respectively. Of the wearing time (median value 842·8 min/d), 35·9 % was accounted for by the PAT and 64·1 % by the SBT.

**Table 3.** Physical activity of all participants and each DP (Medians and percentiles)

			DP1 Various foods					DP2 Red meats and coffee					DP3 Bread and processed meats				
Variables	All participants		Low-trend group		High-trend group		*P*	Low-trend group		High-trend group		*P*	Low-trend group		High-trend group		*P*
	Median	25th–75th percentile	Median	25th–75th percentile	Median	25th–75th percentile		Median	25th–75th percentile	Median	25th–75th percentile		Median	25th–75th percentile	Median	25th–75th percentile	
Subjective-measured PA (*n* 998)																	
PAI, MET ×h/week	15·9	8·7–26·3	13·9	7·4–25·7	17·0	9·8–26·8	<0·01	15·7	8·8–27·0	16·0	8·7–25·5	0·93	15·1	8·4–26·7	16·3	9·0–25·6	0·64
PAT, min/d	330·0	190·0–521·3	300·0	180·0–510·0	350·0	210·0–535·0	0·01	330·0	180·0–540·0	330·0	200·8–510·0	0·84	330·0	190·0–540·0	330·0	200·0–510·0	1·00
SBT, h/d	2·5	1·5–4·0	2·5	1·5–4·0	2·5	1·5–4·0	0·05	2·5	1·5–4·0	2·5	1·5–4·0	0·19	2·5	1·5–4·0	2·5	1·5–4·0	0·42
Objective-measured PA (*n* 914)																	
PAI, MET × h/week	21·1	18·9–23·3	20·6	18·4–22·9	21·7	19·3–23·8	<0·001	21·0	19·1–23·7	21·2	18·9–23·0	0·24	21·0	19·0–23·2	21·3	19·0–23·2	0·69
PAT, min/d	309·1	240·7–375·8	291·9	226·8–358·4	324·2	255·8–395·2	<0·001	307·2	247·4–384·6	311·3	236·0–371·4	0·23	307·0	240·6–376·6	309·4	242·2–375·4	0·77
PAT, % of wearing time	35·9	29·4–44·3	34·8	28·2–42·5	37·7	30·9–45·6	<0·001	36·3	29·9–44·5	35·7	28·8–44·2	0·33	36·1	29·1–44·9	35·6	29·9–43·7	0·47
SBT, min/d	534·3	464·8–605·2	537·9	466·3–612·3	532·0	460·3–593·7	0·04	533·1	462·4–602·7	535·7	465·9–606·1	0·56	533·0	457·1–597·4	535·0	471·1–610·3	0·10
SBT, % of wearing time	64·1	55·7–70·6	65·2	57·5–71·8	62·3	54·4–69·1	<0·001	63·7	55·5–70·1	64·3	55·8–71·2	0·33	63·9	55·1–70·9	64·4	56·4–70·1	0·47

PAI; physical activity index, PAT; physical activity time, SBT; SB time, DP; dietary pattern. The effective number of participants is shown next to the item; values are shown as median (25th–75th percentile), and the *P*-value is a test of the difference between the high- and low- trend groups for each dietary pattern; a low- or high- trend group means Scoring lower or higher than the median on the principal component score. the intensity of accelerometer measurements of 1.5 METs or less was regarded as SB, and 1.6 METs or more was considered PA. PAI in the subjective measure is the sum of walking and exercise (METs × h/week), and PAI in the objective measure is the sum of PA (METs × h/week).

Table 4 shows the associations between the DP and the subjective-measure PA. After adjusting for other variables, few significant associations were observed between each DP and outcomes, although DP1 ‘various foods’ was negatively associated with the SBT (B (partial regression coefficient): −0·16; 95 % CI −0·30, 0·02).

**Table 4.** The relationship between dietary patterns and subjective-measured physical activity (95 % confidence intervals)

	PAI, MET × h/week						PAT, min/d						SBT, h/d					
	Model 1			Model 2			Model 1			Model 2			Model 1			Model 2		
	B	95 % CI	*P*	B	95 % CI	*P*	B	95 % CI	*P*	B	95 % CI	*P*	B	95 % CI	*P*	B	95 % CI	*P*
DP1 Various foods (*n* 881)	0·95	–0·11, 2·02	0·08	0·71	–0·35, 1·77	0·19	14·96	–5·48, 35·30	0·15	10·59	–9·66, 30·84	0·31	–0·22	–0·36, 0·08	<0·01	–0·16	–0·30, 0·02	0·02
DP2 Red meats and coffee (*n* 881)	0·004	–1·06, 1·06	0·99	–0·10	–1·13, 0·93	0·85	–6·60	–26·82, 13·62	0·52	–8·49	–28·17, 11·19	0·40	–0·07	–0·21, 0·07	0·34	–0·09	–0·22, 0·05	0·22
DP3 Bread and processed meats (*n* 881)	–0·34	–1·39, 0·72	0·53	–0·23	–1·26, 0·80	0·66	–4·29	–24·39, 15·80	0·68	–1·22	–20·85, 18·41	0·90	0·10	–0·04, 0·24	0·14	0·12	–0·02, 0·26	0·09

B; Partial regression coefficient, CI; Confidence interval, PA; physical activity, PAI; PA index, PAT; PA time, DP; dietary pattern. Model 1 was adjusted for sex, age, BMI, and MMSE. Model 2 was adjusted for ADL, WHO-5, years of education, living status, economic status, smoking habit, medical history, and need for long-term care in addition to the variables in Model 1. PAI in the subjective measure is the sum of walking and exercise (METs × h/week), and PAT is the sum of walking and exercise time (h/d).

Table 5 shows the association between DP and PA measured by accelerometers. PAI was significantly negatively associated with DP3 ‘bread and processed meats’ (B: −0·20; 95 % CI −0·33, −0·06). PAT was positively associated with DP1 ‘various foods’ (B: 6·30; 95 % CI 0·34, 12·59) and negatively associated with DP2 ‘red meats and coffee’ (B: −7·20; 95 % CI −13·34, −1·05) and DP3 ‘bread and processed meats’ (B: −8·40; 95 % CI −14·28, −2·51). In contrast to PAT, SBT was negatively associated with DP1 ‘various foods’ and positively associated with DP2 ‘red meats and coffee’ and DP3 ‘bread and processed meats’.

**Table 5.** The relationship between dietary patterns and objective-measured physical activity (95 % confidence intervals)

	PAI, MET × h/week						PAT, min/d						SBT, min/d					
	Model 1			Model 2			Model 1			Model 2			Model 1			Model 2		
	B	95 % CI	*P*	B	95 % CI	*P*	B	95 % CI	*P*	B	95 % CI	*P*	B	95 % CI	*P*	B	95 % CI	*P*
DP1 Various foods (*n* 809)	0·11	–0·04, 0·25	0·14	0·10	–0·05, 0·24	0·19	6·46	0·34, 12·59	0·04	6·30	0·18, 12·42	0·04	–6·46	–12·59, 0·34	0·04	–6·30	–12·42, −0·18	0·04
DP2 Red meats and coffee (*n* 809)	–0·14	–0·28, −0·003	0·06	–0·13	–0·26, −0·01	0·08	–7·20	–13·34, −1·05	0·02	–6·52	–12·55, −0·50	0·03	7·20	1·05, −13·34	0·02	6·52	0·50, 12·55	0·03
DP3 Bread and processed meats (*n* 809)	–0·20	–0·33, 0·06	<0·01	–0·16	–0·29, −0·03	0·02	–8·40	–14·28, −2·51	<0·01	–6·92	–12·72, −1·13	0·02	8·40	2·51, 14·28	<0·01	6,92	1·13, 12·72	0·02

B; Partial regression coefficient, CI; Confidence interval, PA; physical activity, PAI; PA index, PAT; PA time, SBT; sedentary behaviour time, DP; dietary pattern. Model 1 was adjusted for sex, age, BMI, MMSE, and wearing time. Model 2 was adjusted for ADL, WHO-5, years of education, living status, economic status, smoking habit, medical history, and need for long-term care in addition to the variables in Model 1. The intensity of accelerometer measurements of 1.5 METs or less was regarded as SB, and 1.6 METs or more was considered PA. PAI in the objective measure is the sum of PA (METs × h/week), and PAT in the objective measure is the sum of PA time (min/d).

## Discussion

This study identified major DP in adults aged ≥ 85 and examined their relationship with PA and SB by a questionnaire and accelerometer. These DP were DP1 ‘various foods’; DP2 ‘red meats and coffee’ and DP3 ‘bread and processed meats’. The method of PA measurement (questionnaire *v*. accelerometer) showed significant differences in the relationship with DP. Objective measurements with accelerometers showed a positive association between DP1 ‘various foods’ and PAT and a relatively negative association with SBT. DP2 ‘red meat and coffee’ showed a negative association with PAI and PAT, and DP3 ‘bread and processed meats’ showed a negative association with PAT, inversely to DP1 ‘various foods’. This is the first study to examine the relationship between DP and PAT and SBT measured by the accelerometer in this age group.

DP are diverse in various populations, and in Japan alone, 285 different DP have been identified by factor analysis or PCA. After examining their similarities, six major DP were finally determined^(31)^. A study in the same age group (≥ 85 years) as the present study was conducted in the UK, and the reported DP were characterised by red meat or butter consumption^(32)^. In this study, the DP1 ‘various foods’ was similar to the DP previously named ‘healthy’ or ‘prudent’. DP2 ‘red meats and coffee’ is identical to that in the same age group in the UK, characterised by red meat intake, and DP3 ‘bread and processed meats’ is similar to the Western pattern. In a study on adults aged ≥ 85 in central Japan, a DP characterised by a high intake of plant-based foods and a high food diversity was positively associated with subjectively measured PAI^(33)^. In Croatia (Zagreb City), using the Elderly Dietary Index Score, ten foods (meat, fish, fruits, vegetables, grains, legumes, olive oil, alcohol, bread and dairy products) were assessed, and their association with PA was examined. Results indicated that ‘optimal’ intakes of meat, seafood, grains, fruits, legumes and bread were associated with ‘adequate’ PA^(24)^. At different ages, in an Asian population (mean age 70 years), the healthy dietary behaviours were determined from six food groups (1) omnivore, (2) milk and dairy products, (3) vegetables, (4) fruits, (5) soya, fish, meat and eggs and (6) nuts, seeds, oils and fats were examined and compared with PA by subjective and objective measurements^(23)^. In the subjective measures, the ‘Healthy Diet’ group had significantly longer total leisure-time PA and walking than the ‘No Healthy Diet’ group. The ‘Healthy Diet’ group had significantly longer light-intensity PAT and shorter SBT as measured objectively. The findings of this study are generally consistent. In the Australian population (48 % men, mean age 60 years) and Netherlands population (56 % men, mean age 62 years), a positive association between fruit and vegetable intake and leisure time PA and PA level was observed, suggesting that the consumption of fruits and vegetables and the implementation of PA significantly impact the health of the older population^(34,35)^. This study showed differences between the association of DP and subjective or objective measures of PA. In particular, they differed in the relationship between DP and subjective measures of PA with and without WHO-5 (data not shown). WHO-5 is a useful indicator of mental health status, and the association between PA and health status has been reported^(36)^. The association between DP and objective-measured PA did not differ significantly between those with and without WHO-5. Therefore, in subjective measures, the significant change could be attributed to the self-reported longer PA of participants with good mental health compared with those without it. The correlation coefficient between the WHO-5 and the subjective-measured PAI was significant at 0·20 (*P* < 0·001).

The relationship between diet and PA behaviours in the older population is evidenced in the frailty cycle. It is unclear, which is more pertinent, but if the decline in activity leads to a decrease in energy requirements, the individual will have a reduced appetite and eat less. This may eventually lead to phenotypic outcomes such as weight loss and loss of muscle mass^(37)^, while it has been argued that PA has the potential to modulate resting hunger and satiety in the older population and may benefit the older population by promoting EBRB. However, increased lean body mass and RMR have been observed but may not necessarily be associated with increased energy intake^(38–40)^. The association between a healthy or high-quality diet and PA could be explained by the participants’ socio-economic status and living status^(41,42)^. Their good condition is associated with greater health awareness, increased knowledge of healthy foods and motivated to do more PA. Environmental factors such as food availability, food quality, transportation, housing type and availability of rest areas may influence EBRB^(43,44)^. In addition, animal studies have reported a reduced appetite for unhealthy foods in high-intensity PA groups. Conversely, some physiological mechanisms may be responsible for choosing unhealthy foods in populations with low PA^(45)^. In this study, the relationship was observed in a previously unobserved population aged ≥ 85, and the tendency towards relatively unhealthy DP was associated with lower PAI and PAT, which may be an essential finding in health interventions for the older population. However, the factors that determine them need to be carefully considered to transform these behaviours. The intake of plant-based foods, such as vegetables and fruits, or the practice of PA benefits sarcopenia and frailty^(46,47)^. Plant foods contain anti-inflammatory components associated with inflammatory markers^(48)^. This food group is also rich in micronutrients such as vitamin C, which has antioxidant properties. They protect against muscle deterioration caused by oxidative stress^(49)^. PA has been reported to play an essential role in inflammatory markers and oxidative stress and may also increase skeletal muscle mass^(50,51)^. Although both are important, it is expected that populations with a high intake of plant-based foods have more PA; thus, it is advocated that the concurrent effects of these factors should be considered when examining health-related outcomes^(8)^. In contrast, the trend towards unhealthy DP was associated with physical inactivity and longer SBT compared with DP1 ‘various foods’; DP2 ‘red meats and coffee’ is a DP with a higher intake of animal products, while DP3 ‘bread and processed meats’ is a DP with a lower food diversity. The relationship between these dietary characteristics and physical function or ADL in the older population has been discussed. However, the effects of physical inactivity and SB are also of interest^(16,52–55)^. As the association between a healthy diet and PA habits has been observed, the influence of PA rather than diet alone may be sufficient, especially for muscle-related outcomes (such as hand grip strength, sarcopenia and frailty). Therefore, a comprehensive examination of these factors may be required. In addition, it is important to consider intervention studies in terms of diet and PA^(56)^. As a sensitivity analysis, participants who identified with potential for dementia (MMSE < 24, *n* 62) were excluded and re-identified for DP to confirm their association with PA, but no significant change was observed. Likewise, as the number of participants in the subjective and objective surveys in the PA was different, the numbers were matched and re-examined, but the level of significance remained unchanged.

This study’s findings help fill the gap in research by suggesting an association between DP and PA in adults aged ≥ 85 that has not been previously examined in the EBRB and HB. In addition, the findings can provide insights for practitioners or researchers of health-promoting behavioural changes, allowing for more effective intervention trials and social implementation and will eventually be helpful for evidence-based policy-making. Previous studies have reported that PA and SB have different effects on the body’s inflammatory response^(19)^. It may not be sufficient to focus only on HB examining the association between DP and PA in combination with health-related outcomes from a behavioural perspective. Therefore, in future careful consideration should be given to identify which behaviour patterns, including unhealthy behaviours, are associated with which health-related outcomes. There is also a need to determine the most accurate method of assessing PA in older people, especially in this age group.

This study has several strengths. First, it focuses on DP, which represent the participants’ usual dietary behaviours, rather than on single nutrients. Second, the study used a linear regression model and considered several important confounders, including demographic and socio-economic variables, lifestyle habits and clinical data. Third, subjective and objective measurements for PA were used, and the differences were also analysed. Fourth, the study included more than 1000 adults aged ≥85, which is the largest worldwide. However, this study also has several limitations. First, this cross-sectional study is observational but not causal, meaning the results cannot be used to determine the cause. Second, the study was limited to Kawasaki City residents who could come to the study site and had low levels of long-term care. Therefore, the results need to be examined longitudinally, considering the study design, to determine the general applicability of these results. In addition, future studies should explore the benefits and impact of implementing healthy diet and PA behaviours.

### Conclusions

This study observed HB implementation in adults aged ≥ 85, suggesting that a trend towards healthier, more food-diverse DP is associated with longer PAT, and a relatively unhealthy diet is associated with shorter PAT and longer SBT. Future studies should examine the outcomes of HB implementation in this age group. When examining health-related outcomes, it may be necessary to comprehensively consider EBRB or HB, not just diet or PA.
